# Role of Pre-operative Inflammatory Markers as Predictors of Lymph Node Positivity and Disease Recurrence in Well-Differentiated Pancreatic Neuroendocrine Tumours: Pancreas2000 Research and Educational Program (Course 9)

**DOI:** 10.3389/fmed.2020.00346

**Published:** 2020-08-11

**Authors:** Lulu Tanno, Antonio Pea, Taina Nykänen, Pooja Prasad, Ceren Tuncer, Giovanni Marchegiani, Stuart Robinson

**Affiliations:** ^1^Department of General Surgery, University Hospital Southampton NHS Foundation Trust, Southampton, United Kingdom; ^2^Pancreas Institute, University Hospital of Verona (AOUI), Verona, Italy; ^3^Department of Surgery, Hyvinkää Hospital, Hyvinkää, Finland; ^4^Freeman Hospital, Newcastle upon Tyne, United Kingdom; ^5^School of Medicine, Koç University Research Centre for Translational Medicine, Istanbul, Turkey

**Keywords:** pancreatic neuroendocrine tumour, inflammatory index, lymph node involvement, disease outcome, survival

## Abstract

Pancreatic neuroendocrine tumours (PNET) is a rare disease and in the absence of metastases, surgical resection is recommended. Key factors affecting survival in PNETs are the stage and grade of the disease, but there is increasing evidence suggesting lymph node involvement is associated with shorter disease-free and overall survival. Ability to predict the likelihood of lymph node involvement at the time of diagnosis would affect surgical decision making in these patients. A systemic inflammatory index such as neutrophil to lymphocyte ratio or platelet to lymphocyte ratio has been associated with poor prognosis in several cancers.

**Method:** This study is a retrospective multi-centre study. The data including pre-operative inflammatory markers such as haemoglobin, neutrophil, lymphocyte counts and pathological data including number of positive lymph nodes, tumour grade and size, are collected to assess the association between inflammatory index and lymph node involvement.

**Conclusion:** This study aims to assess the value of routinely available pre-operative haematological markers in predicting lymph node involvement in non-functioning PNETs.

## Background and Rationale for the Study

Pancreatic neuroendocrine tumour (PNET) is a rare disease and comprises of around 3% of newly diagnosed pancreatic malignancies each year (1). In the absence of metastatic disease, the European Neuroendocrine Tumour Society (ENETS) guideline recommends curative surgical resection in non-functioning PNETs (2). Deciding the most appropriate surgical approach depends on the size and location of the tumours, disease grade and stage, and patient preference and fitness. Several papers have demonstrated that the key factors affecting survival in PNETs are stage and grade (3–5), however, emerging evidence suggests that lymph node involvement is associated with shorter disease-free and overall survival (6, 7).

The accurate prediction of lymph node involvement at the time of diagnosis substantially impacts the surgical decision making as enucleation or local resection may not be appropriate. The currently available biochemical index, such as chromogranin A, does not provide information on the nodal status. Pre-operative radiology is currently the main method of predicting pre-operative lymph node involvement (8).

A systemic inflammatory index such as neutrophil to lymphocyte ratio (NLR), monocyte to lymphocyte ratio (MLR), or platelet to lymphocyte ratio (PLR) has been associated with poor prognosis in several cancers (9, 10). Studies have demonstrated an association between high NLR and MLR, and poor overall and recurrence free survival in resected PNETs (11, 12). A study by Zhou et al. suggests that NLR of 1.8 is associated with lymph node metastasis (13), however, there is currently limited evidence to support this finding of inflammatory index and nodal metastasis. Haematological values are therefore not routinely used in clinical practice to predict lymph node involvement.

This study aims to assess the value of routinely available pre-operative haematological markers in predicting lymph node involvement in non-functioning PNETs.

## Objectives and Outcome Measures

### Primary Objective

The primary objective is to assess whether raised pre-operative inflammatory markers are associated with lymph node involvement in low to intermediate grade PNETs by performing a large multi-centre retrospective analysis.

### Secondary Objective

The secondary objective is to compare progression-free survival and overall survival stratified by pre-operative inflammatory markers.

### Outcome Measure

The primary endpoint of this study is to determine the association of systemic inflammatory values (NLR, MLR, and PLR) and the incidence of lymph node involvement. The secondary endpoint is to assess the impact of these ratios on disease-free survival and overall survival.

### Study Design

The study is a retrospective multi-centre cohort study. High volume surgical institutions with recognition and interest in the management of PNET patients (such as ENETs accredited centres of excellence) are invited to take part in this study. A minimum of 20 patients is required to participate. We have received significant interests from several centres and anticipate around 800 patients to be recruited to the study. All the patient data will be anonymised and stored in a database according to good clinical practice (GCP). Individual centres participating in this study are advised to register the study with the appropriate department within their institution.

### Patients Eligibility Criteria

The study includes patients with resected non-functioning grade 1 and grade 2 PNETs.

#### Inclusion Criteria

- Patients undergoing curative surgical resection with lymphadenectomy
- Confirmed diagnosis of well-differentiated pancreatic neuroendocrine tumours on histology
- Non-functional tumours only
- Grade 1 (low) and grade 2 (intermediate) tumours based on ki67 or mitotic index
- The availability of haematological and biochemical blood results either pre-operatively or at the time of diagnosis with no evidence of infection or systemic inflammatory response such as pyrexia, tachycardia, positive blood cultures, or any concurrent infective condition.

#### Exclusion Criteria

- Pre-operative chemotherapy
- Those with a history of cancer of any type
- Grade 3 disease based on Ki67 or mitotic index on histology report
- Poorly differentiated or neuroendocrine carcinoma on histology
- Functional tumours
- Those with confirmed metastatic disease at the time of diagnosis
- Evidence of infection such as pyrexia, systemic inflammatory response, pancreatitis, cholecystitis, jaundice, cholangitis, and other inflammatory condition at the time of diagnosis or the time of haematological testing
- Patients with systemic chronic inflammatory conditions such as inflammatory bowel disease, rheumatoid arthritis, or any condition requiring steroids or systemic anti-inflammatory treatment.

### Study Data

A multinational multi-centre database will be created to collect and collate data from the medical records of participating institutions.

### Recruitment

Allocated data supporting medical personnel, appointed by the institutions to collect the data for this study, will collect anonymised data on:

- Demographic details (age, sex), presenting symptoms, significant comorbidities, findings from pre-operative cross-sectional imaging, and laboratory findings including chromogranin A, haemoglobin, total white blood cell count, neutrophil count, lymphocyte count, monocyte count, and CRP (when available).
- Details of surgery and pathology results.
- We will also ask each centre to provide their biochemical and haematological normal range values as this may vary according to centres.

#### Participant Identification

Each centre will have different procedures. At University hospital Southampton, there is an existing database on all patients diagnosed with neuroendocrine tumours. Similar databases would also exist for other European neuroendocrine tumour society accredited centres of excellence. This database would be used to identify patients.

People who would be involved in inputting the data, would be those of service users that already have access to the data, or clinical staff members. However, no patient identifiable information will be recorded in the database for the study.

### Statistics and Data Analysis

All the data analysis will be performed by the Pancreas 2000 project members using IBM SPSS version 25 for windows and Microsoft excel windows version 10.

#### Demographics Analysis

Categorical data will be presented as proportions; continuous data will be presented as either mean (standard deviation) or median (interquartile range) as appropriate. The difference between the ratios and the clinic-pathological features will be analysed using the *t*-test if parametric or the Mann-Whitney U-test if non-parametric.

#### Primary Endpoint Analysis

Receiver operative characteristic (ROC) analysis will be performed to identify the predictive cut-off points for the different ratios. The association between clinical, pathological, and inflammatory ratios will be analysed using the univariate and multivariate analyses.

#### Secondary Endpoint Analysis

Disease-free survival and overall survival will be analysed using the Kaplan-Meier method and the log-rank test. A *p* < 0.05 will be considered statistically significant.

#### Data Management

The data management plan for this project is publicly accessible from https://dmponline.dcc.ac.uk/. The project is titled “Role of pre-operative inflammatory markers as predictors of lymph node positivity and disease recurrence in well-differentiated pancreatic neuroendocrine tumours.”

## Dissemination Policy

### Deadline for Participation and Data Collection

The last Pancreas2000 course 9 meeting will take place in November 2020 by which time data analysis must be completed. Therefore, we ask each participating institution to submit their data to the lead coordinator of this project (LT l.tanno@soton.ac.uk) by September 2020.

### Authorship and Publication Policy

Authorship will be based on the recommendations from the international committee of medical journal editors (ICMJE). The first five authors will be the members of the Pancreas 2000 participants of this project (LT, AP, PP, CT, and TN). The last two authorship positions are reserved for the two mentees of this group (GM and SR). All other authors will be listed in alphabetical order. Two authors will be listed as co-authors from participating institutions, provided a minimum of 20 patients have been recruited into the study. If the institution contributes more than 50 patients to this study, additional co-authorships will be allocated.

## Ethics Statement

The studies involving human participants were reviewed and approved by the Health Research Authority Ethics committee UK (REC reference 20/LO/0219). Written informed consent from the [patients/participants OR patients/participants legal guardian/next of kin] was not required to participate in this study in accordance with the national legislation and the institutional requirements.

## Author's Note

This protocol has regard for the Health Research Authority (HRA) guidance Integrated Research Application System (IRAS) Number: 268529 University Hospital Southampton (UHS) Sponsors Number: RHM CAN1550.

## Author Contributions

The protocol was written jointly between LT, AP, TN, CT, and PP as part of the pancreas2000 study group. LT had been the main author who has been editing the protocol and updated the information in order to adhere to the publishing guidelines for this journal. The study group was supervised by GM and SR and they have provided advice in terms of reviewing the protocol and suggesting areas of amendments to make the study more clear. All authors contributed to the article and approved the submitted version.

## Conflict of Interest

The authors declare that the research was conducted in the absence of any commercial or financial relationships that could be construed as a potential conflict of interest.
